# Dendritic Cell-Derived TSLP Negatively Regulates HIF-1α and IL-1β During Dectin-1 Signaling

**DOI:** 10.3389/fimmu.2019.00921

**Published:** 2019-05-08

**Authors:** Matthew J. Elder, Steve J. Webster, Timothy J. Fitzmaurice, Aran S. D. Shaunak, Martin Steinmetz, Ronnie Chee, Ziad Mallat, E. Suzanne Cohen, David L. Williams, J. S. Hill Gaston, Jane C. Goodall

**Affiliations:** ^1^Department of Medicine, School of Clinical Medicine, Addenbrookes Hospital, University of Cambridge, Cambridge, United Kingdom; ^2^Early Oncology R&D Division, AstraZeneca, Cambridge, United Kingdom; ^3^Department of Veterinary Medicine, University of Cambridge, Cambridge, United Kingdom; ^4^Unit 970, INSERM, Paris Cardiovascular Research Center, Paris, France; ^5^Department of Immunology, Royal Free Hospital, London, United Kingdom; ^6^Division of Cardiovascular Medicine, Department of Medicine, University of Cambridge, Cambridge, United Kingdom; ^7^Biopharmaceutical Research Division, AstraZeneca, Cambridge, United Kingdom; ^8^Department of Surgery, Center for Inflammation, Infectious Disease and Immunity, James H. Quillen College of Medicine, East Tennessee State University, Johnson City, TN, United States

**Keywords:** TSLP, dectin-1, IL-1β, hypoxia, ROS, HIF-1α, Syk

## Abstract

Thymic stromal lymphopoietin (TSLP) is a functionally pleotropic cytokine important in immune regulation, and TSLP dysregulation is associated with numerous diseases. TSLP is produced by many cell types, but has predominantly been characterized as a secreted factor from epithelial cells which activates dendritic cells (DC) that subsequently prime T helper (T_H_) 2 immunity. However, DC themselves make significant amounts of TSLP in response to microbial products, but the functional role of DC-derived TSLP remains unclear. We show that TSLPR signaling negatively regulates IL-1β production during dectin-1 stimulation of human DC. This regulatory mechanism functions by dampening Syk phosphorylation and is mediated via NADPH oxidase-derived ROS, HIF-1α and pro-IL-1β expression. Considering the profound effect TSLPR signaling has on the metabolic status and the secretome of dectin-1 stimulated DC, these data suggest that autocrine TSLPR signaling could have a fundamental role in modulating immunological effector responses at sites removed from epithelial cell production of TSLP.

## Introduction

Thymic Stromal Lymphopoietin (TSLP) is a four-helix bundle cytokine belonging to the IL-2 family that was initially described as a lymphocyte growth factor (1). Since this initial report, it has been shown to be produced by a plethora of cell types (2–8). Functionally TSLP is pleotropic; TSLP is described to have an important role in maintaining tolerance within the gut (9, 10) yet it is implicated in asthma (11) and in the skin in both the development of itch (12) and atopic inflammation (12–14). TSLP binds to its unique receptor, called the TSLP receptor (TSLPR) composed of a unique TSLPR chain and the IL-7 receptor alpha (15) which initiates JAK-STAT mediated activation of downstream target genes (16–18).

Dendritic cells (DC) are immunologically important TSLP responsive cells (19). DC activated with TSLP can induce naïve CD4^+^ T cell proliferation (20) and T helper (T_H_) 2-cell differentiation (21) which requires the up-regulation of OX40L on the DC (22). DC can also produce TSLP in response to pattern recognition receptor (PRR) engagement (3, 4, 8, 23). Therefore, DC are the only cell population known to both produce and respond to TSLP by altering their effector responses. However, the functional role of DC-derived TSLP remains unclear; this work addresses this issue.

Amongst PRRs, dectin-1 stimulation induces TSLP production by DC (8, 23). Dectin-1 recognizes exposed β-1,3 glucan residues on the cell surface of fungi and studies utilizing dectin-1 gene knockout (^−/−^) mice emphasize the importance of this PRR to anti-fungal immunity (24). Activation of dectin-1 signaling induces immunological effector responses including phagocytosis (25), oxidative burst (26, 27) and the secretion of inflammatory cytokines including IL-1β, IL-6, and IL-23 (27–32). IL-1β production plays a critical role in the generation of protective anti-fungal immunity (33, 34). However, IL-1β dysregulation is associated with numerous diseases including inflammatory bowel disease (IBD) and auto-inflammatory conditions such as the cryopyrin associated periodic syndromes (CAPS) (35, 36). Therefore, IL-1β production is tightly regulated in DC, requiring two independent signals for its production. An initial priming signal (signal 1) is generated from ligation of PRRs, activating the inflammatory transcription factor NF-κB required for the up-regulated transcription of pro-IL-1β (36). A second activatory signal (signal 2) then causes inflammasome-mediated cleavage of pro-IL-1β into its active form (26, 27, 30, 32, 36–39). Recent work has augmented the understanding of IL-1β regulation, describing how changes to cellular metabolism after PRR stimulation regulate IL-1β expression (40, 41).

We report here that autocrine TSLPR signaling in human DC negatively regulates IL-1β production in response to dectin-1 stimulation. It likely does this through limiting a metabolic switch to glycolysis in DC which is required for IL-1β expression.

## Results

### Inhibition of TSLPR Signaling in mDC Modulates IL-1β Production

**Figure 1:**
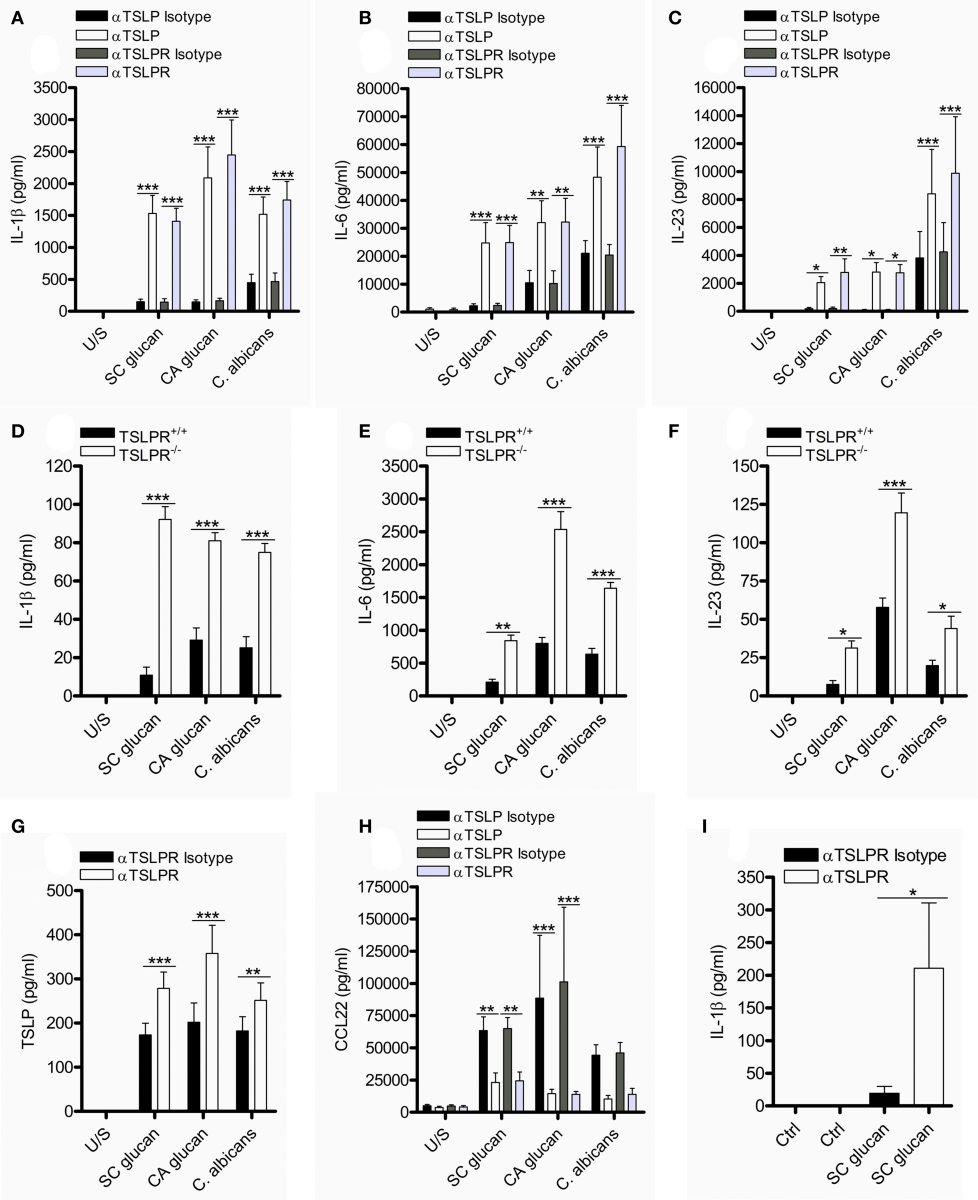
Inhibition of TSLPR signaling in mDC modulates IL-1β production. **(A–C,G,H)** Human mDC stimulated with SC glucan, CA glucan or heat killed *C. albicans* hyphae with anti-TSLP, anti-TSLPR or IgG isotype control antibodies for 24 h (*n* = 14 independent donors, presented as pooled data for SC glucan stimulated mDC), (*n* = 6 independent donors, presented as pooled data for CA glucan stimulated mDC) and (*n* = 10 independent donors, presented as pooled data for heat killed *C. albicans* hyphae stimulated mDC). **(D–F)** Wildtype TSLPR^+/+^ or TSLPR^−/−^ BMDC derived from BALB/c mice were stimulated with, SC glucan, CA glucan or *C. albicans* hyphae for 24 h (*n* = 4 independent animals from a representative experiment, presented as pooled data. Experiment was repeated four times). **(I)** Human *ex vivo* CD1c^+^ DC stimulated with SC glucan with anti-TSLPR or IgG isotype control antibodies for 24 h (*n* = 3 independent donors, presented as pooled data). IL-1β, IL-6, IL-23, TSLP and CCL22 was measured in 24-h cell culture supernatants by ELISA. Cumulative data displayed as mean ±SEM. Statistical analysis calculated using one-way ANOVA with Bonferroni post-tests (^***^p = 0.001, ^**^p = 0.01, ^*^p = 0.05).

DC secretion of TSLP can be readily induced following dectin-1 stimulation (4, 8, 23). We investigated the functional relevance of DC-derived TSLP by neutralizing TSLP signaling in human monocyte-derived dendritic cells (mDC) which had been stimulated with heat-killed *C. albicans* or β-glucan purified from either *S. cerevisiae* (SC glucan) or *C. albicans* (CA glucan). We blocked TSLP activity or TSLPR signaling using neutralizing antibodies, and evaluated IL-1β, IL-6, IL-23, TSLP, and CCL22 secretion. Inhibition of either TSLP or TSLPR on mDC augmented the production of IL-1β, IL-6, IL-23 (Figures 1A–C), and TSLPR inhibition augmented TSLP itself (Figure 1G) from mDC in response to dectin-1 stimulation. These effects were not observed using control antibodies. CCL22 is a known TSLP-responsive chemokine in epithelial cells (21), and as expected inhibition of TSLPR signaling also reduced DC CCL22 production in response to dectin-1 stimulation (Figure 1H). Augmented IL-1β, IL-6, and IL-23 were all dependent on signaling through dectin-1 via Syk (Supplemental Figures 1A–F). To ensure that this effect was not an artifact caused by antibody binding and subsequent mDC activation, we generated bone marrow-derived dendritic cells (BMDC) from wildtype TSLPR^+/+^ and knockout TSLPR^−/−^ BALB/c mice. In agreement with the results using TSLPR blocking antibodies, TSLPR^−/−^ BMDC stimulated with either *C. albicans* or β-glucans produced more IL-1β, IL-6, and IL-23 compared to TSLPR^+/+^ BMDC (Figures 1D–F). Furthermore, inhibiting TSLPR signaling on human blood-derived CD1c^+^ DC also increased IL-1β secretion confirming that these findings were not an artifact of *in vitro* differentiation of monocytes (Figure 1I).

### TSLPR Signaling Negatively Regulates IL-6 and IL-23 Secretion by Controlling IL-1β

**Figure 2:**
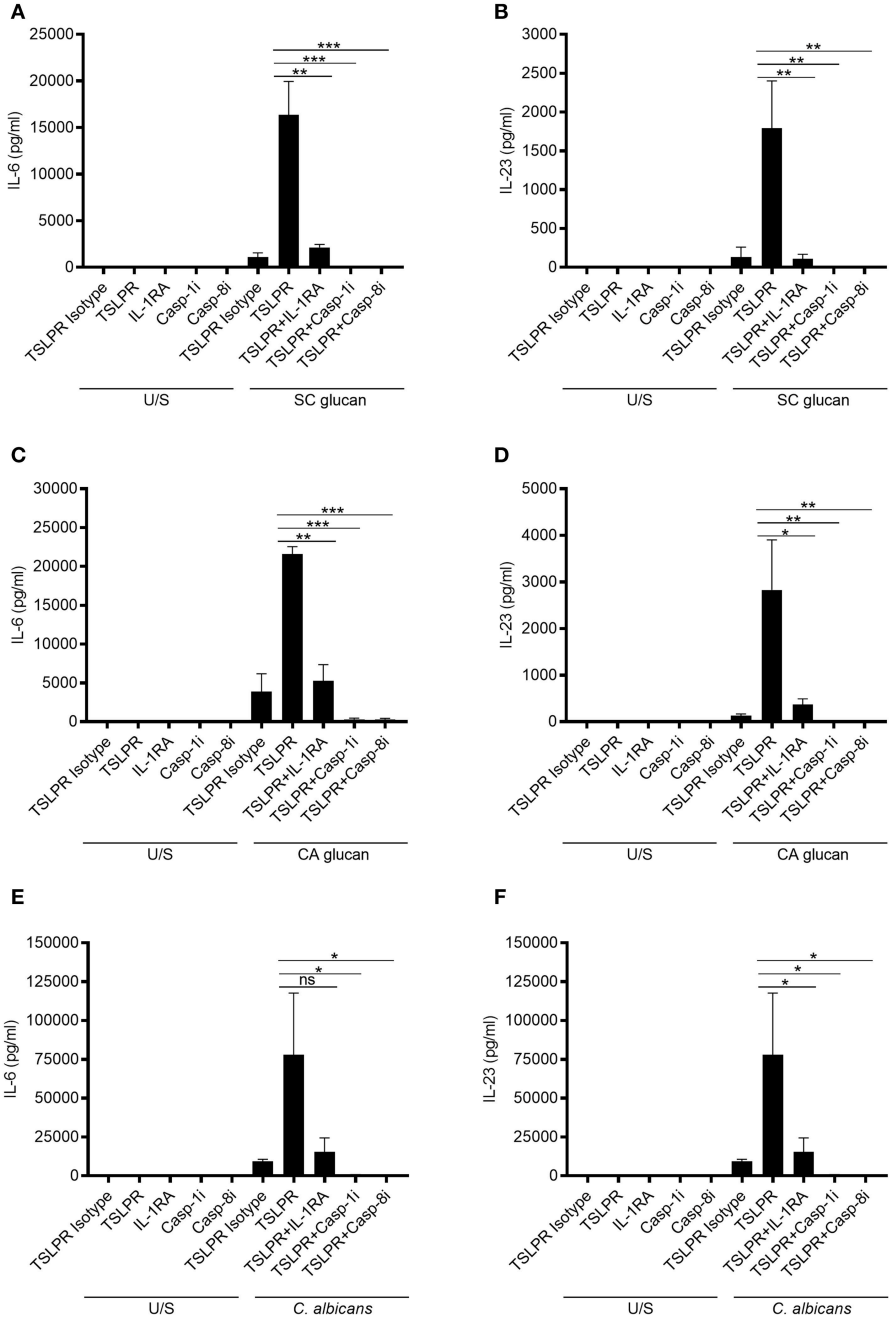
mDC-derived TSLP indirectly regulates IL-6 and IL-23 secretion by controlling IL-1β production. Human mDC stimulated with, **(A,B)** SC glucan, **(C,D)** CA glucan or **(E,F)** heat killed *C. albicans* hyphae with anti-TSLPR or IgG isotype control antibodies in the presence or absence of IL-RA, caspase-1 inhibitor or caspase-8 inhibitor for 24 h (*n* = 3 independent donors, presented as pooled data). IL-6 and IL-23 was measured in 24-h cell culture supernatants by ELISA. Cumulative data displayed as mean ±SEM. Statistical analysis calculated using one-way ANOVA with Bonferroni post-tests (ns = not significant, ^***^p = 0.001, ^**^p = 0.01, ^*^p = 0.05).

The importance of IL-1β for generating effective anti-fungal immunity is well-established (31). Therefore, we wanted to determine whether the increase in IL-6 and IL-23 secretion from mDC was a direct effect of inhibiting TSLPR signaling on production of these cytokines or due to the effects of increased IL-1β. We have showed that IL-1β mRNA expression precedes that of IL-6 and IL-23p40 (Supplemental Figures 2A–D) and inhibition of IL-1 receptor signaling with IL-1 receptor antagonist (IL-1RA) significantly inhibits IL-6 and IL-23 secretion from SC glucan-stimulated mDC (Supplemental Figures 2E, F). However, to directly address this, we stimulated mDC with *C. albicans* or β-glucans and neutralized TSLPR signaling in the presence or absence of IL-1RA, or caspase-1 and caspase-8 inhibitors to prevent inflammasome-mediated processing of IL-1β which our group has previously demonstrated (8). This showed that the increased IL-6 and IL-23 observed when TSLPR was blocked, was reduced in the presence of IL-1RA or caspase-1 and caspase-8 inhibitors (Figures 2A–F). Therefore, autocrine TSLPR signaling indirectly regulated IL-6 and IL-23 secretion by its effect on IL-1β production.

### Dectin-1-Induced TSLP Negatively Regulates Pro-IL-1β and HIF-1α

**Figure 3:**
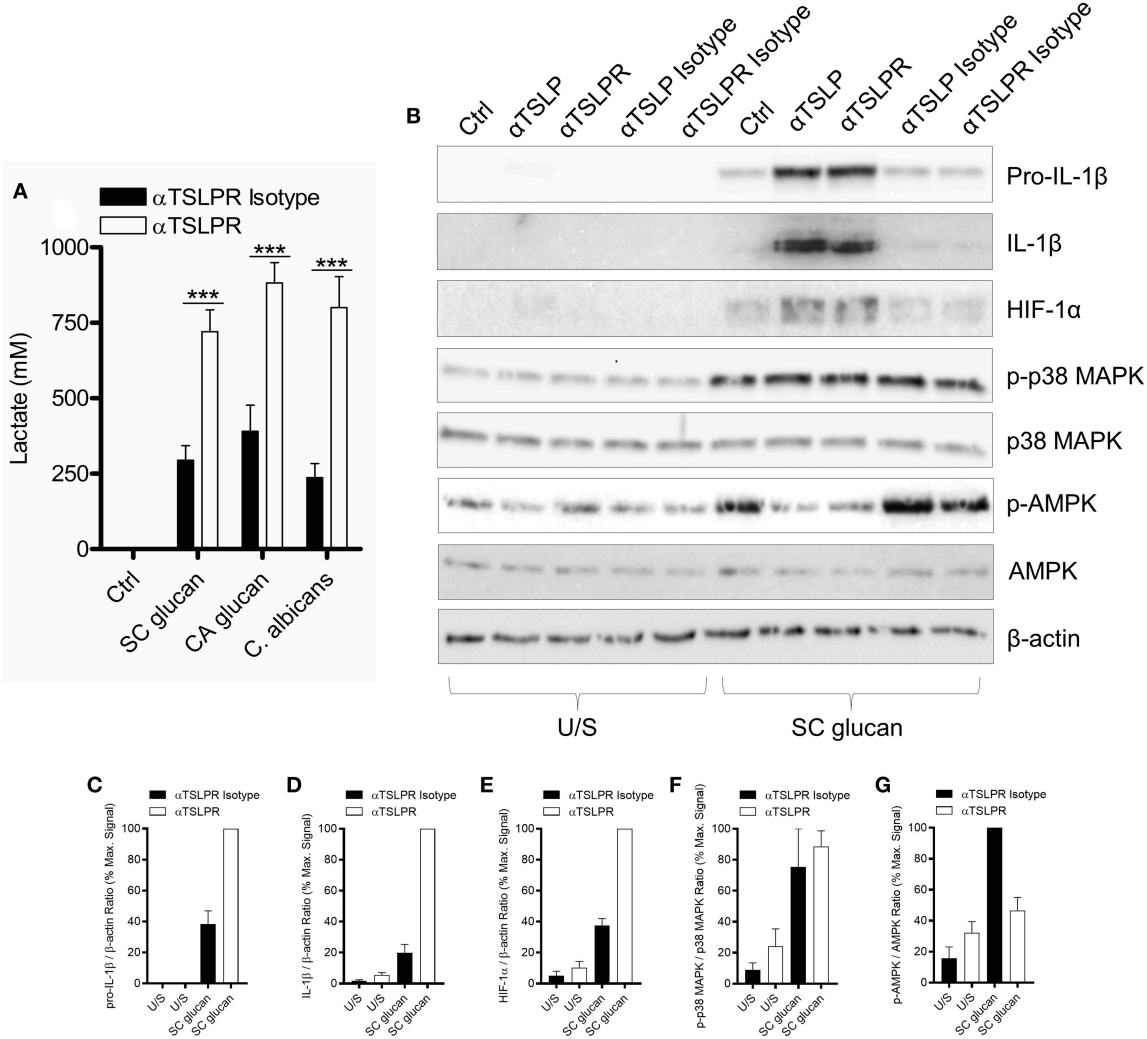
Dectin-1-induced TSLP negatively regulates pro-IL-1β and HIF-1α. **(A)** Human mDC were stimulated with SC glucan, CA glucan or heat killed *C. albicans* hyphae with anti-TSLPR antibodies or IgG isotype control for 24 h (*n* = 6 independent donors, presented as pooled data). Lactate production was measured in cell-culture supernatants using colourmetric L-lactate detection kit. **(B)** Human mDC were stimulated SC glucan with either anti-TSLP, anti-TSLPR or IgG isotype control antibodies for 8 h (*n* = 1 representative donor presented, three separate experiments performed). Pro-IL-1β, IL-1β, HIF-1α, phospho-p38 MAPK, p38 MAPK, phospho-AMPK, AMPK and β-actin were measured by immunoblot. **(C–G)** Densitometry of cumulative data was performed using Image Studio Lite software with pro-IL-1β, IL-1β and HIF-1α normalized to β-actin and phospho-p38 MAPK and phospho-AMPK normalized to total p38 MAPK and AMPK respectively. Data is reported as percentage of maximal signal observed within each donor (*n* = 3 independent donors, presented as pooled data). Cumulative data displayed as mean +SEM. Statistical analysis calculated using one-way ANOVA with Bonferroni post-tests (^***^p = 0.001).

**Figure 4:**
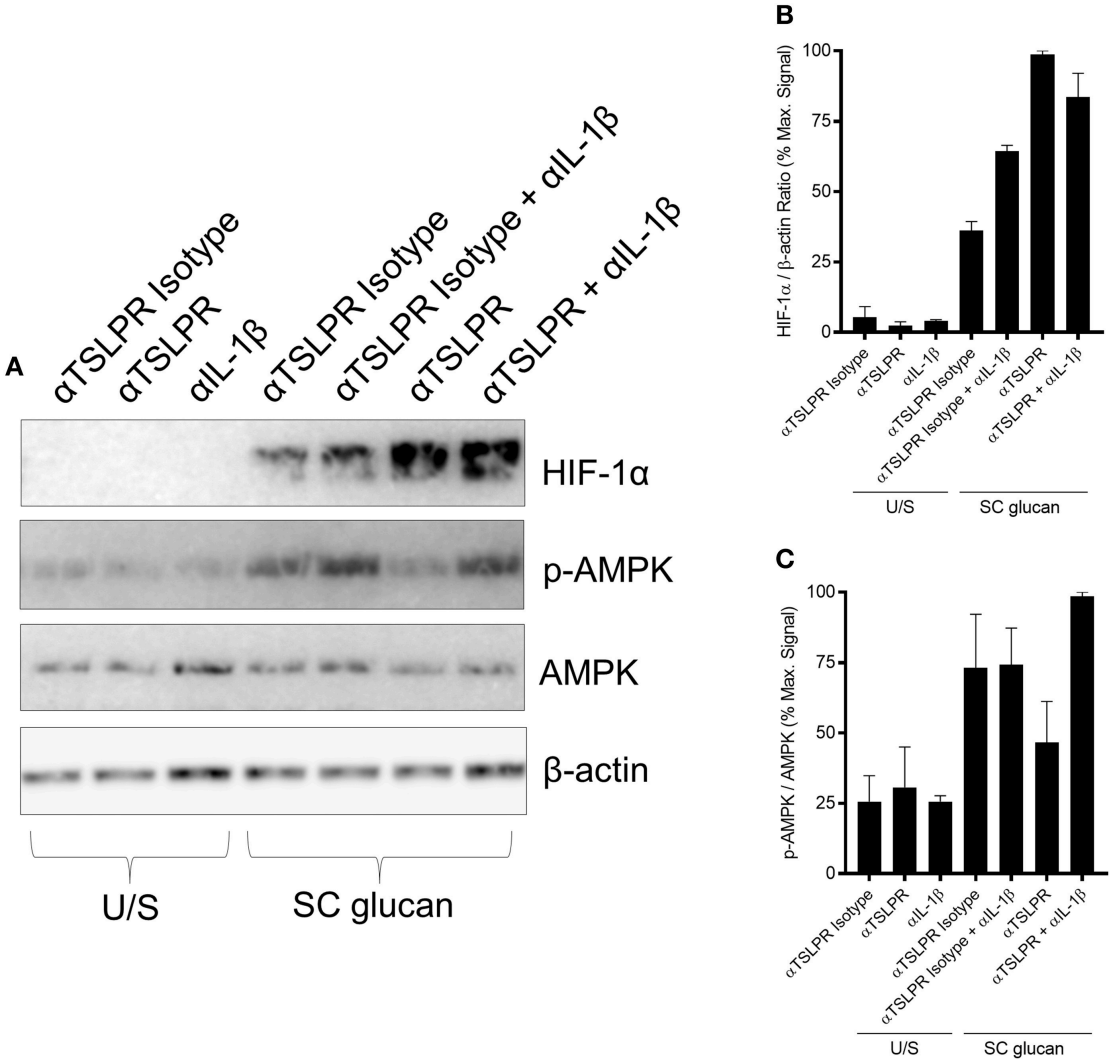
Increased HIF-1α expression is regulated by TSLPR inhibition and not IL-1β secretion **(A)** Human mDC were stimulated with SC glucan with either anti-TSLPR or IgG isotype control antibodies for 8 h in the presence or absence of IL-1β neutralization antibodies (*n* = 1 representative donor presented, three separate experiments performed). Pro-IL-1β, IL-1β, HIF-1α, phospho-p38 MAPK, p38 MAPK, phospho-AMPK, AMPK and β-actin were measured by immunoblot. **(B,C)** Densitometry of cumulative data was performed using Image Studio Lite software with HIF-1α normalized to β-actin and phospho-AMPK normalized to AMPK. Data is reported as percentage of maximal signal observed within each donor (*n* = 3 independent donors, presented as pooled data). Cumulative data displayed as mean +SEM.

Recent work has described how LPS-treated macrophages and β-glucan-stimulated monocytes undergo a metabolic switch toward glycolysis and away from oxidative phosphorylation (40). This is a process which has many parallels with the Warburg effect observed in tumors. We observed that mDC culture media was more acidic when TSLPR signaling was neutralized during dectin-1 stimulation. Therefore, we measured lactate production to determine whether increased lactic acid production accounted for the pH change. Significantly higher concentrations of lactate were indeed detected in cell culture supernatants from mDC when TSLPR signaling was neutralized (Figure 3A) and from TSLPR^−/−^ BMDC (Supplemental Figure 3A), suggesting that autocrine mDC-derived TSLP negatively regulates this metabolic shift to lactate production. Tannahill et al reported that this cellular metabolic switch was crucial for pro-IL-1β expression via the induction of the transcription factor, hypoxia-inducible factor 1-alpha (HIF-1α), and in agreement with this report TSLP or TSLPR neutralization in mDC during dectin-1 signaling augmented both HIF-1α and pro-IL-1β protein expression (Figures 3B–E). An identical augmentation was observed in TSLPR^−/−^ BMDC (Supplemental Figure 3B). The enhancement in HIF-1α expression could not be explained by changes in gene expression since HIF-1α mRNA expression was not significantly modulated by inhibition of TSLPR signaling. In contrast pro-IL-1β mRNA expression was augmented in mDC when TSLPR activity was neutralized (Supplemental Figures 4A,B). These effects were specific and not as a result of a general increase in expression of effector molecules downstream of dectin-1 signaling; for instance p38 mitogen-activated protein kinase (MAPK) activation (Thr 180/Tyr 182) was not modulated by TSLP or TSLPR neutralization (Figures 3B,F). It has previously been shown that chemicals which induce AMP-activated protein kinase (AMPK) activation can oppose the metabolic switch in DCs and macrophages which is induced by PRR stimulation (41). Accordingly, neutralization of TSLPR signaling reduced phosphorylation of Thr 172 on the catalytic subunit of AMPK, a key modification required for AMPK activation (Figures 3B,G). To determine whether the modulation of HIF-1α expression and AMPK phosphorylation occurred as a result of the increased IL-1β expression, IL-1β activity was neutralized in combination with inhibition of TSLPR signaling. This showed that the modulation of AMPK activation by inhibition of TSLPR activity was dependent on IL-1β signaling, but this did not apply to HIF-1α expression (Figures 4A–C).

### Dectin-1-Induced TSLP Limits IL-1β, HIF-1α Expression, Syk Phosphorylation and the Activation of NADPH Oxidase-Derived ROS

**Figure 5:**
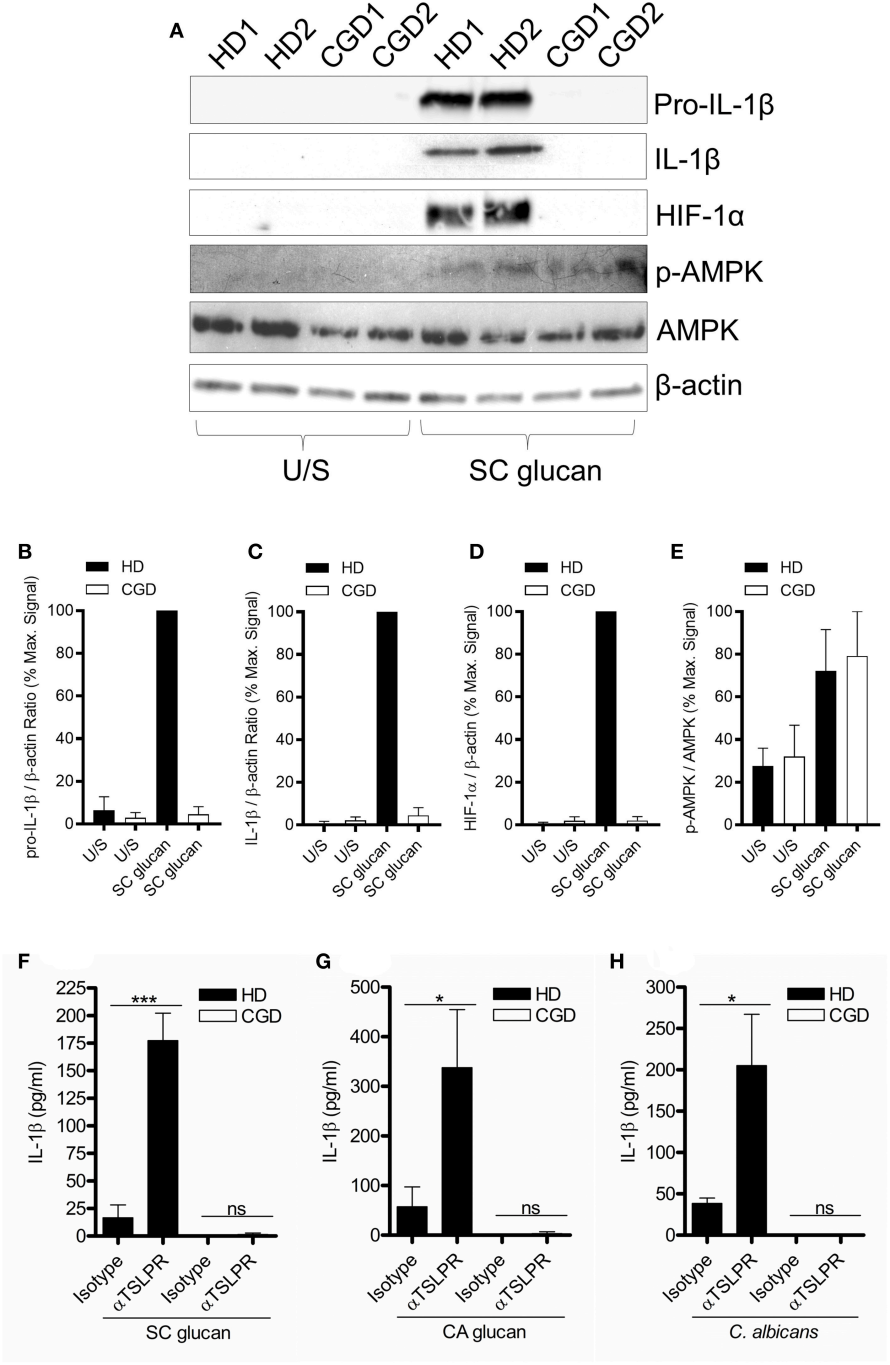
Dectin-1-induced NADPH oxidase-derived ROS is required for augmented IL-1β during TSLPR inhibition. **(A)** Human mDC derived from healthy donors (HD) or CGD patients were stimulated with SC glucan for 8 h (*n* = 2 representative donors presented, three separate experiments performed). Pro-IL-1β, IL-1β, HIF-1α, phospho-AMPK, AMPK and β-actin were measured by immunoblot. **(B–E)** Densitometry of cumulative data was performed using Image Studio Lite software with pro-IL-1β, IL-1β and HIF-1α normalized to β-actin and phospho-AMPK normalized to total AMPK. Data is reported as percentage of maximal signal observed within each donor (*n* = 3 independent donors, presented as pooled data). **(F–H)** Human mDC derived from HD or CGD patients were stimulated with SC glucan, CA glucan or heat killed *C. albicans* hyphae with anti-TSLPR or IgG isotype control antibodies for 24 h (*n* = 3 independent donors, presented as pooled data). IL-1β was measured in 24-h cell culture supernatants by ELISA. Cumulative data displayed as mean +SEM. Statistical analysis calculated using one-way ANOVA with Bonferroni post-tests (^***^p = 0.001,^*^p = 0.05).

It has previously been shown that reactive oxygen species (ROS) are induced in macrophages during dectin-1 signaling and are important for IL-1β production (26, 27). Chronic granulomatous disease (CGD) patients possess mutations in genes encoding proteins that form the nicotinamide adenine dinucleotide phosphate-oxidase (NADPH) complex; thus these patients are unable to generate NADPH oxidase-derived ROS (Supplemental Figure 5) and TSLP from mDC in response to dectin-1 agonists (23). mDC from CGD donors did not show induction of HIF-1α and pro-IL-1β in response to dectin-1 signaling, highlighting the critical role of ROS in the expression of HIF-1α and pro-IL1β by DCs (Figures 5A–D). In contrast, induction of AMPK phosphorylation was unaffected by the absence of a functional NADPH oxidase (Figures 5A,E), indicating that AMPK activation has distinct signaling from that required for HIF-1α expression. Given the important role of ROS in HIF-1α protein expression, we investigated whether inhibition of TSLPR signaling in CGD patients modulated IL-1β production. TSLPR neutralization during dectin-1 stimulation did not alter the minimal amount of IL-1β secretion seen in CGD donors (Figures 5F–H). Our data suggest NADPH oxidase-derived ROS contributes to the enhancement of HIF-1α and IL-1β expression observed with the loss of TSLPR signaling.

### Dectin-1-Induced TSLP Negatively Regulates Syk Activation

**Figure 6:**
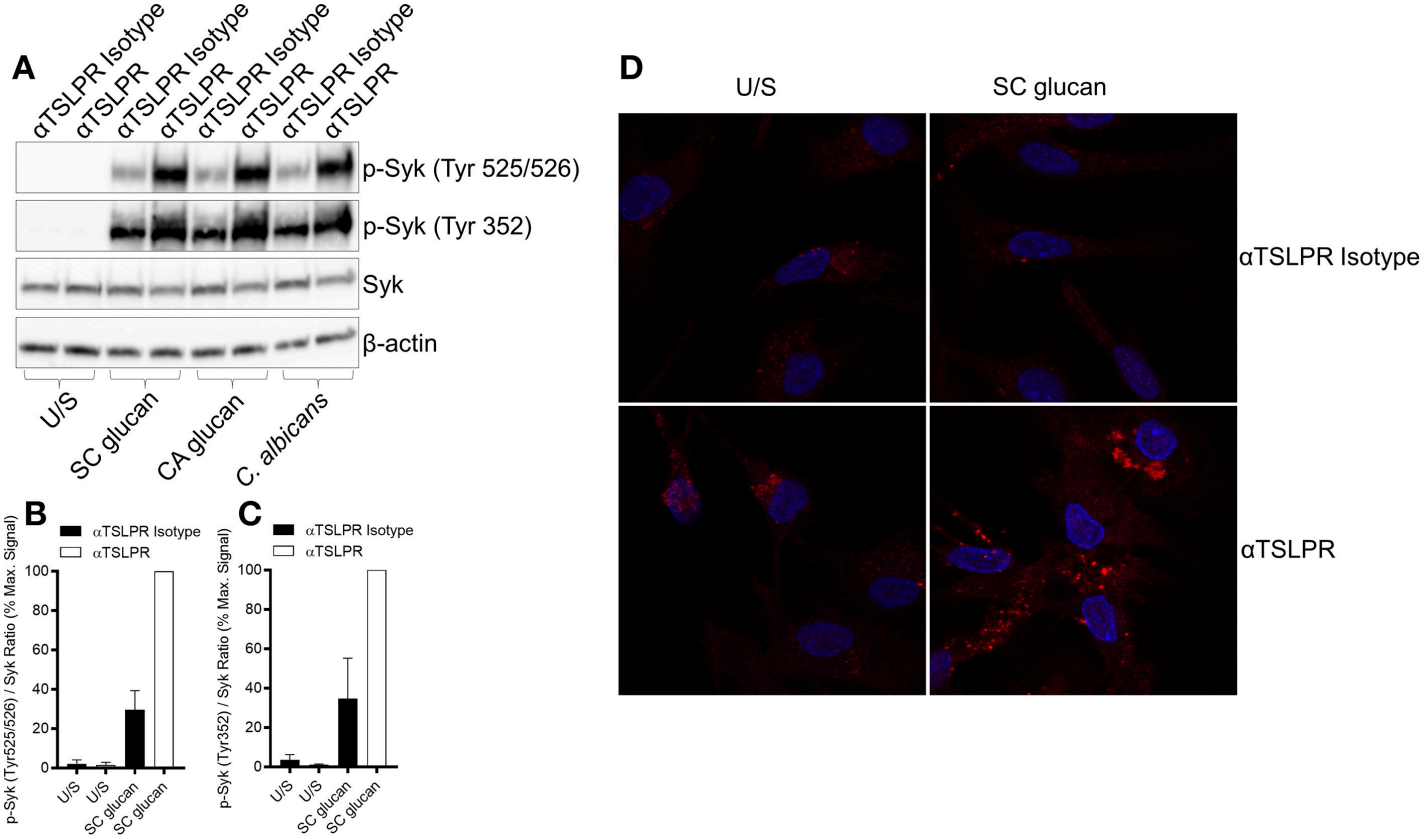
Dectin-1-induced TSLP negatively regulates Syk activation. **(A–D)** Human mDC were stimulated with SC glucan with anti-TSLPR or IgG isotype control antibodies for 2 h (*n* = 1 representative donor presented, three separate experiments performed). **(B,C)** Densitometry of cumulative data was performed using Image Studio Lite software with phospho-Syk normalized to total Syk. Data is reported as percentage of maximal signal observed within each donor (*n* = 3 independent donors, presented as pooled data). Phospho-Syk measured by **(A)** immunoblot and **(D)** confocal microscopy (Red represents p-Syk and Blue represents nuclear DAPI staining).

Dectin-1-mediated effector responses are controlled by the recruitment and activation of Syk (28), and we have previously shown that inhibition of Syk signaling in mDC stimulated with dectin-1 agonists substantially reduces TSLP and IL-1β expression (8). Furthermore, inhibition of Syk activity in DCs with the Syk inhibitor R406 reduced SC glucan-induced HIF-1α and pro-IL-1β expression (Supplemental Figure 6A). We hypothesized that TSLPR signaling may directly modulate Syk activation and therefore examined the phosphorylation status of critical Tyr residues in Syk which are associated with its activation and interaction with downstream signaling pathways. As expected, SC glucan-induced Syk phosphorylation (Tyr 525/526) was dectin-1 dependent (Supplemental Figure 6B). Neutralization of autrocrine TSLPR signaling resulted in enhanced Syk phosphorylation at this residue (Figures 6A–D). Furthermore, analysis of the phosphorylation state of other Tyr residues associated with Syk activation was also enhanced (Figures 6A,C). These data suggest that autocrine mDC-derived TSLP limits Syk-mediated activation. This in turn may negatively regulate the metabolic shift to glycolysis, the production of HIF-1α and hence expression of pro-IL-1β.

## Discussion

We have previously demonstrated that human mDC and murine BMDC generate TSLP in response to *C. albicans* or β-glucans (8, 23). In this study, we showed that this TSLP acts in an mDC autocrine fashion to regulate IL-1β, and hence IL-6 and IL-23 production. We propose that this increase in inflammatory cytokine expression which is seen when DC responses to TSLP are blocked is a result of enhanced HIF-1α expression and a more marked glycolytic shift in the metabolism of the DC. Furthermore, we showed that TSLPR signaling dampens Syk phosphorylation likely acting to decrease HIF-1α and pro-IL-1β production.

TSLPR signaling negatively regulates IL-1β production, which in turn modulates the expression of IL-6 and IL-23. It is well-established that IL-1β production plays a critical role in the generation of protective anti-fungal immunity (31); however, IL-1β dysregulation is associated with IBD and CAPS such as Muckle-Wells syndrome (35, 36). Given the importance of IL-1β regulation, we speculate that DC-derived TSLP acts as an important molecular checkpoint to limit IL-1β-mediated effector responses. Furthermore, the differentiation of naïve CD4^+^ T cells to T_H_1- and T_H_17-cells is important for protective anti-fungal immunity and the inflammatory cytokines IL-1β, IL-6, and IL-23 are important in generating these T cell phenotypes (28, 31, 42–44). TSLPR^−/−^ mice have been shown to produce more IFN-γ in an experimental modal of *Trypanosoma congolense* infection (45), more IFN-γ and IL-17 in an inducible modal of colitis (46) compared to TSLPR^+/+^ mice and IL-1β is crucial for the generation of inflammatory IFN-γ/IL-17 double producing T cells during *C. albicans* infection (31, 42). Given that autocrine TSLPR signaling negatively regulated IL-1β production during *C. albicans* and β-glucan stimulation: TSLPR signaling might also function to regulate T_H_1- and T_H_17-cell differentiation.

Recent work has established that myeloid-derived cells stimulated with activators of PRRs switch from oxidative phosphorylation to aerobic glycolysis (40). Similarly, we observed that *C. albicans* and β-glucan stimulated mDC also induced HIF-1α and increased the production of lactate. Furthermore, both HIF-1α and lactate production were further augmented when TSLPR signaling was neutralized on dectin-1-stimulated mDC. Given that Tannahill et al identified that HIF-1α expression was crucial for pro-IL-1β induction in LPS-treated macrophages (40) and β-glucan stimulated monocytes induce HIF-1α expression required for this glycolytic switch (41): these data are compatible with the idea that autocrine TSLPR signaling controls pro-IL-1β expression in mDC by regulating HIF-1α. Further work will be required to confirm that HIF-1α is a key factor that promotes increased IL-1β expression when TSLPR signaling is inhibited.

IL-1β has been shown to directly induce HIF-1α expression (47–49), but despite an increase in IL-1β when inhibiting TSLPR signaling, our data provide evidence that the enhanced HIF-1α expression induced by dectin-1 occurred independently of this cytokine. In contrast to the regulation of HIF-1α, modulation of AMPK phosphorylation was shown to be dependent on the secretion of IL-1β and to our knowledge this is the first report that suggests that IL-1β may negatively regulate AMPK activation. Our data also highlighted the differences in the signaling requirements for ROS in AMPK activation and HIF-1α expression. We showed that following dectin-1 stimulation, phosphorylation of the AMPK catalytic subunit was unaffected by the absence of ROS but in contrast, HIF-1α expression was completely dependent on ROS production. Given that AMPK activation has been shown to antagonize HIF-1α expression, it may not be surprising that the signaling requirements for these factors have shared and distinct arms, highlighting the potential for independent regulation of these factors in the dectin-1 signaling pathway. Most importantly, the modulation of HIF-1α and AMPK in the presence of TSLPR neutralizing antibodies, could be interpreted as complimentary responses, since enhanced HIF-1α or a reduction in AMPK activation have been shown to contribute to a metabolic shift toward aerobic glycolysis and the increased production of inflammatory cytokines in monocytes and DC (41, 50).

Our data also showed that inhibition of TSLPR signaling on mDC enhanced Syk activation. We show that CGD patients cannot generate ROS, HIF-1α or IL-1β secretion; and unlike in healthy donors, mDC-derived from CGD patients do not augment HIF-1α expression or IL-1β secretion when autocrine TSLPR signaling is neutralized. These data are in agreement with published work showing that both Syk and ROS regulate IL-1β cleavage during dectin-1 stimulation (27) and that ROS can activate HIF-1α expression (51). Therefore, autocrine TSLP production may directly regulate HIF-1α and pro-IL-1β, via Syk-mediated activation of NADPH oxidase-derived ROS: a regulatory mechanism that does not exist in CGD patients.

Therefore, dysregulation of TSLP production or TSLPR signaling might be a feature of diseases associated with Syk and IL-1β overproduction. Opportunistic invasive fungal infections present serious clinical complications particularly in immunosuppressed individuals. Given the importance of IL-1β to anti-fungal immunity these findings describe an important regulatory mechanism of IL-1β that could ultimately lead to the development of approaches to boost resistance.

## Materials and Methods

### Ethics Statement

Human blood was sourced from apheresis cones derived from healthy donors (HD) (Addenbrooke's Hospital, Cambridge) and age- and sex-matched CGD patients (Royal Free Hospital, London). Appropriate consent to use blood-derived cells for research was obtained. These studies were approved by the Joint UCL/UCLH Committee for the Ethics of Human Research, project number 04/Q0501/119.

### Cell Isolation and Generation of Dendritic Cells

Human monocyte-derived dendritic cells (mDC) were generated from CD14^+^ monocytes isolated from human PBMC by magnetic bead separation (Miltenyi) and were differentiated by culturing for 6-days in RPMI1640 (Lonza) 5% FCS (Biosera) supplemented with 20 ng/ml GM-CSF (Life Technologies) and 4 ng/ml IL-4 (BD Biosciences) as described previously (8). CGD donors were recruited at the Royal Free Hospital, London and age and sex matched to healthy donors recruited at the University of Cambridge, in accordance with ethical rules set out by each institution. Human CD1c^+^ DC were isolated from PBMC by magnetic bead separation (Miltenyi). Murine bone marrow-derived dendritic cells (BMDC) were generated by culturing cells isolated from bone marrow of wildtype (TSLPR^+/+^) and TSLPR knockout (TSLPR^−/−^) BALB/c mice for 7-days in RPMI1640 10% FCS supplemented with 5% X63 conditioned media and 10 ng/ml IL-4 (Peprotech).

### Cell Stimulations

mDCs were stimulated with 50 μg/ml of either β-1,3 glucan (SC glucan) derived from *Saccharomyces cerevisiae* (*S. cerevisiae*) isolated by David. L. Williams, East Tennessee State University as previously described (52), β-1,3 glucan (CA glucan) derived from hyphal *Candida albicans* (*C. albicans*) isolated by David. L. Williams as previously described (53) or heat-killed hyphal *C. albicans* (MOI 2:1) gifted from John Trowsdale, University of Cambridge. *C. albicans* was grown in sabouraud dextrose broth for 8 h at 37°C to an optical density of 0.2. *C. albicans* was killed by heating for 1 h at 70°C.

### Reagents

10 μg/ml sheep anti-TSLP blocking antibody (R&D Systems), 10 μg/ml sheep IgG isotype control (R&D Systems), 10 μg/ml goat anti-TSLPR blocking antibody (R&D Systems), 10 μg/ml goat IgG isotype control (R&D Systems), 2 μg/ml IL-1β blocking antibody (R&D Systems), 10 μg/ml mouse IgG_2B_ dectin-1 blocking antibody (clone-259931 R&D Systems), 10ug/ml mouse IgG_2B_ isotype control (clone-20116 R&D Systems), 1 μM Syk inhibitor, R406 (Selleckchem), 1 μg/ml IL-1 receptor antagonist (IL-1RA) (R&D Systems), 50 μM caspase-1 inhibitor (Z-YVAD-FMK) (Calbiochem), 50 μM caspase-8 inhibitor (Z-IE(OMe)TD(OMe)-FMK) (Calbiochem). Where inhibitors, blocking antibodies and modifiers were used, mDCs were pre-treated 1 h prior to cell stimulation. Repeated experiments were performed on independent donors unless otherwise stated.

### Cytokine Production

IL-1β, IL-6, IL-23 (eBioscience), TSLP, and CCL22 (R&D) were measured in 24 h mDC, CD1c^+^ DC or BMDC culture supernatants by ELISA according to manufacturer's protocols.

### Quantitative Real-Time PCR

mDC were stimulated for indicated time period and IL-1β, HIF-1α, IL-6, IL-23p19 and IL-23p40 mRNA expression was measured by quantitative real-time PCR from isolated RNA (Norgen) using TaqMan Gene Expression Assays (Applied Biosystems). Gene expression was normalized to HPRT and calculated as relative expression (2-dCT).

### Immunoblot

mDC were stimulated for indicated time period and protein lystates were generated, quantified by Bradford assay (Thermo) and resolved using SDS-PAGE. IL-1β (R&D, AB-201-AB), HIF-1α (Novus, NB100-449), phospho-AMPK (Thr 172) (Cell Signaling, 2535), AMPK (Cell Signaling, 5831), phospho-Syk (Tyr 525/526) (Cell Signaling, 2710), phospho-Syk (Tyr 352) (Cell Signaling, 2701), Syk (Cell Signaling, 13198), phospho-p38 MAPK (Thr 180/ Tyr 182) (Cell Signaling, 4511), p38 MAPK (Cell Signaling, 8690) and β-actin (Abcam, 8226) protein expression were measured by immunoblot, by incubation with indicated primary antibodies followed by incubation with HRP-conjugated secondary antibodies, ECL detection (PerkinElmer) and visualized using GBox (Syngene). Densitometry of cumulative data was performed using Image Studio Lite software. Pro-IL-1β, IL-1β and HIF-1α expression was normalized to β-actin and phospho-p38 MAPK, phospho-AMPK and phospho-Syk was normalized to total p38 MAPK, AMPK and Syk respectively. Cumulative data is reported as percentage of maximal signal observed within each donor.

### Quantification of Reactive Oxygen Species (ROS) Production

mDC derived from HD or CGD patients were stimulated with SC glucan and ROS production was measured by fluorescence of the luminol-based chemiluminescent probe L-012 (WAKO) over 30 min using a luminometer (Centro LB960, Berthold).

### Confocal Microscopy

mDC were stimulated for indicated time period on poly-D-lysine coated coverslips (BD), washed with cold PBS and stained for phospho-Syk (Tyr 525/526) (Cell Signaling, 2710). Coverslips were then mounted on slides with DAPI fluoromount G (Southern Biotech) and analyzed by confocal microscopy (Leica SP5).

### Lactate Detection

mDC were stimulated and lactate production was measured instantly from 24 h cell-culture supernatants using colourmetric L-lactate detection kit (Abcam) according to manufacturer's protocols.

### Data Analysis

Data were analyzed using GraphPad Prism statistical package. Cumulative data are displayed as mean ±SEM. Statistical analysis using either t test (*p*-values stated in figures legends) or one-way ANOVAs with Bonferroni post-tests ns = not significant, ^*^p < 0.05, ^**^p < 0.01, ^***^p < 0.001.

## Ethics Statement

Human blood was sourced from apheresis cones derived from healthy donors (Addenbrooke's Hospital, Cambridge) and age and sex-matched CGD patients (Royal Free Hospital, London). Appropriate consent to use blood-derived cells for research was obtained. Ethics Reference Number: 04/Q0501/119.

## Author Contributions

ME designed, performed and analyzed all experimental data and drafted the manuscript. SW, ZM, EC, JSG, and JCG were key to experimental design, data interpretation, and reviewed manuscript. TF, AS, and MS performed and analyzed experimental data. RC facilitated access to patient blood and aided data interpretation and reviewed manuscript. DW facilitated access to β-glucan agonists and aided experimental design, data interpretation, and reviewed manuscript.

### Conflict of Interest Statement

ME and EC are employed by AstraZeneca. The remaining authors declare that the research was conducted in the absence of any commercial or financial relationships that could be construed as a potential conflict of interest.

## Abbreviations

TSLP: Thymic stromal lymphopoietin
mDC: monocyte-derived DC
BMDC: bone marrow-derived dendritic cell.
