# Supplementary material for: Dendritic Cell-Derived TSLP Negatively Regulates HIF-1α and IL-1β During Dectin-1 Signaling
Source: Front Immunol. 2019 May 8;10:921. doi: 10.3389/fimmu.2019.00921 (PMC6519317; doi:10.3389/fimmu.2019.00921)
Supplement: Supplementary file 1 [file Presentation_1.pptx]

## Slide 1
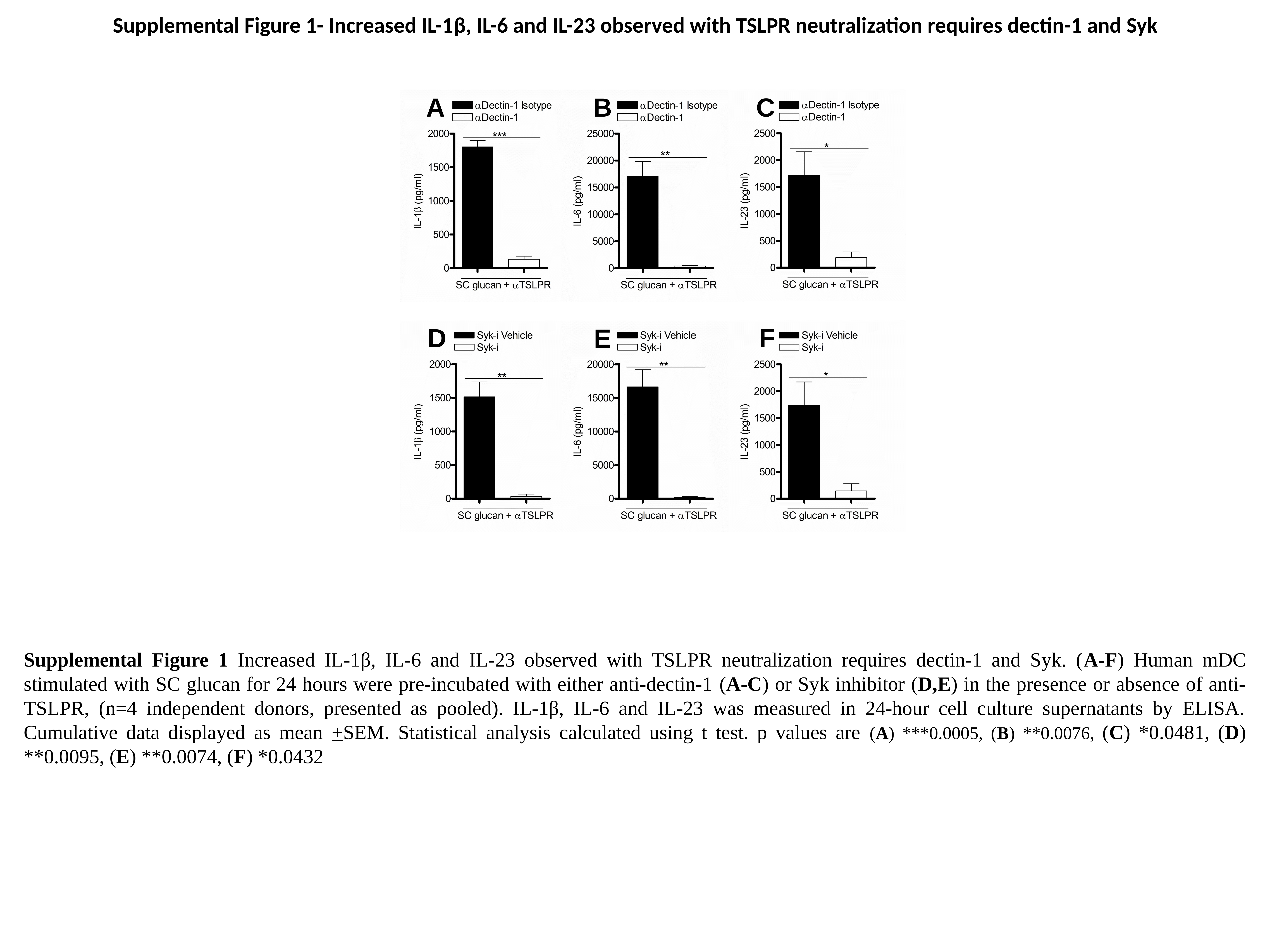

Supplemental Figure 1- Increased IL-1β, IL-6 and IL-23 observed with TSLPR neutralization requires dectin-1 and Syk
A
B
C
F
D
E
Supplemental Figure 1 Increased IL-1β, IL-6 and IL-23 observed with TSLPR neutralization requires dectin-1 and Syk. (A-F) Human mDC stimulated with SC glucan for 24 hours were pre-incubated with either anti-dectin-1 (A-C) or Syk inhibitor (D,E) in the presence or absence of anti-TSLPR, (n=4 independent donors, presented as pooled). IL-1β, IL-6 and IL-23 was measured in 24-hour cell culture supernatants by ELISA. Cumulative data displayed as mean +SEM. Statistical analysis calculated using t test. p values are (A) ***0.0005, (B) **0.0076, (C) *0.0481, (D) **0.0095, (E) **0.0074, (F) *0.0432

## Slide 2
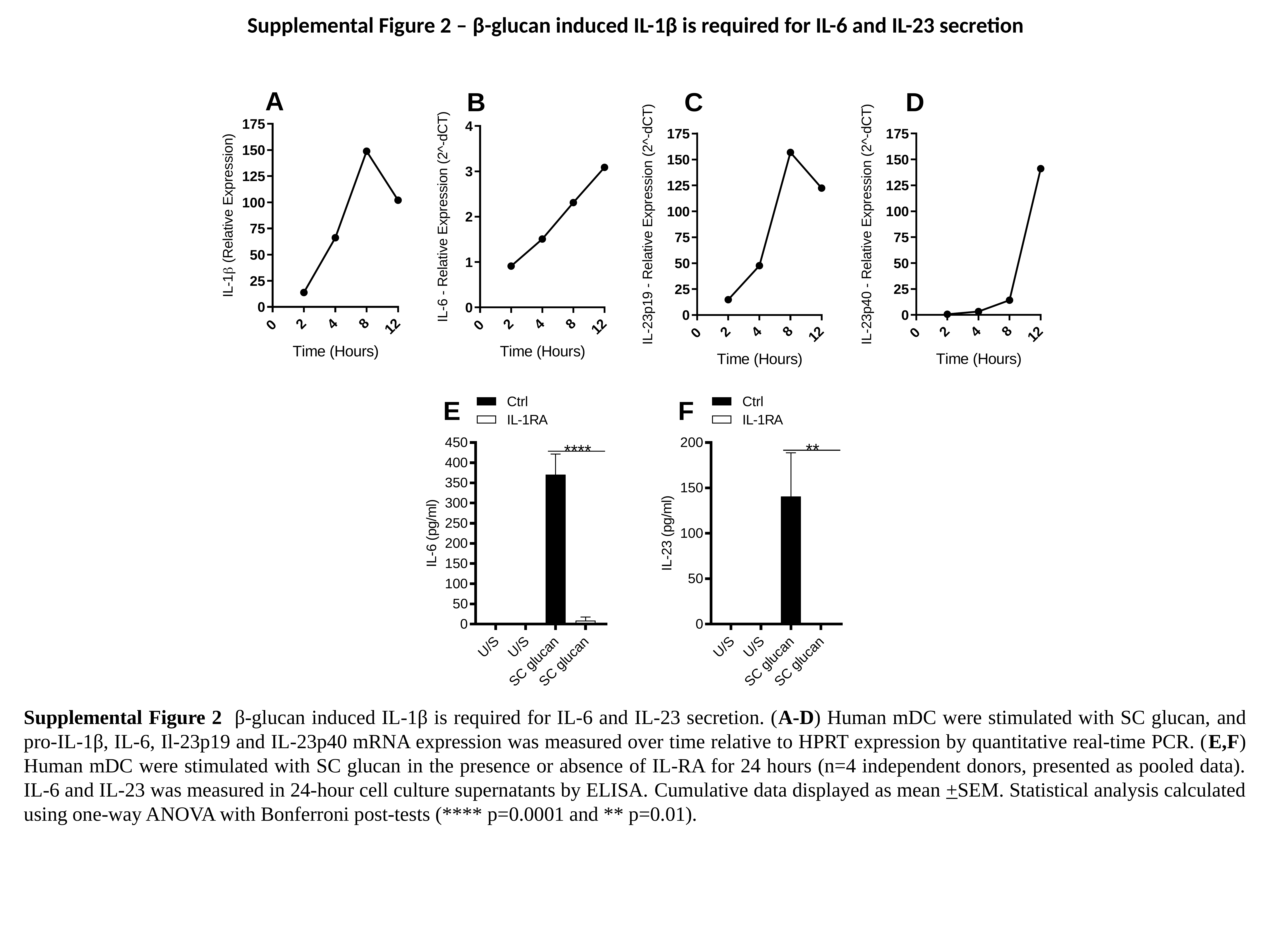

Supplemental Figure 2 – β-glucan induced IL-1β is required for IL-6 and IL-23 secretion
A
B
C
D
E
F
Supplemental Figure 2 β-glucan induced IL-1β is required for IL-6 and IL-23 secretion. (A-D) Human mDC were stimulated with SC glucan, and pro-IL-1β, IL-6, Il-23p19 and IL-23p40 mRNA expression was measured over time relative to HPRT expression by quantitative real-time PCR. (E,F) Human mDC were stimulated with SC glucan in the presence or absence of IL-RA for 24 hours (n=4 independent donors, presented as pooled data). IL-6 and IL-23 was measured in 24-hour cell culture supernatants by ELISA. Cumulative data displayed as mean +SEM. Statistical analysis calculated using one-way ANOVA with Bonferroni post-tests (**** p=0.0001 and ** p=0.01).

## Slide 3
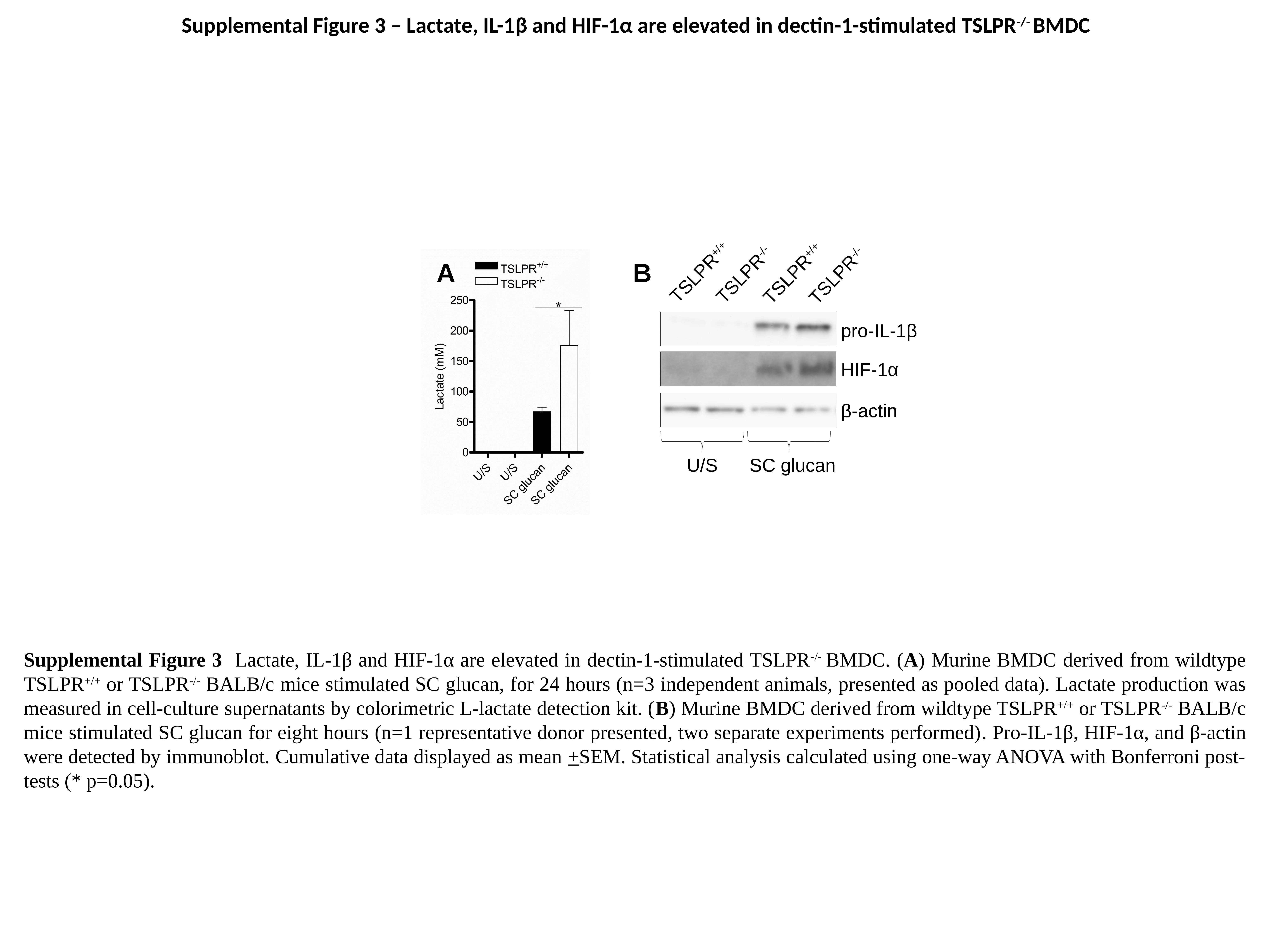

Supplemental Figure 3 – Lactate, IL-1β and HIF-1α are elevated in dectin-1-stimulated TSLPR-/- BMDC
TSLPR+/+
TSLPR+/+
TSLPR-/-
TSLPR-/-
pro-IL-1β
HIF-1α
β-actin
U/S
SC glucan
B
A
Supplemental Figure 3 Lactate, IL-1β and HIF-1α are elevated in dectin-1-stimulated TSLPR-/- BMDC. (A) Murine BMDC derived from wildtype TSLPR+/+ or TSLPR-/- BALB/c mice stimulated SC glucan, for 24 hours (n=3 independent animals, presented as pooled data). Lactate production was measured in cell-culture supernatants by colorimetric L-lactate detection kit. (B) Murine BMDC derived from wildtype TSLPR+/+ or TSLPR-/- BALB/c mice stimulated SC glucan for eight hours (n=1 representative donor presented, two separate experiments performed). Pro-IL-1β, HIF-1α, and β-actin were detected by immunoblot. Cumulative data displayed as mean +SEM. Statistical analysis calculated using one-way ANOVA with Bonferroni post-tests (* p=0.05).

## Slide 4
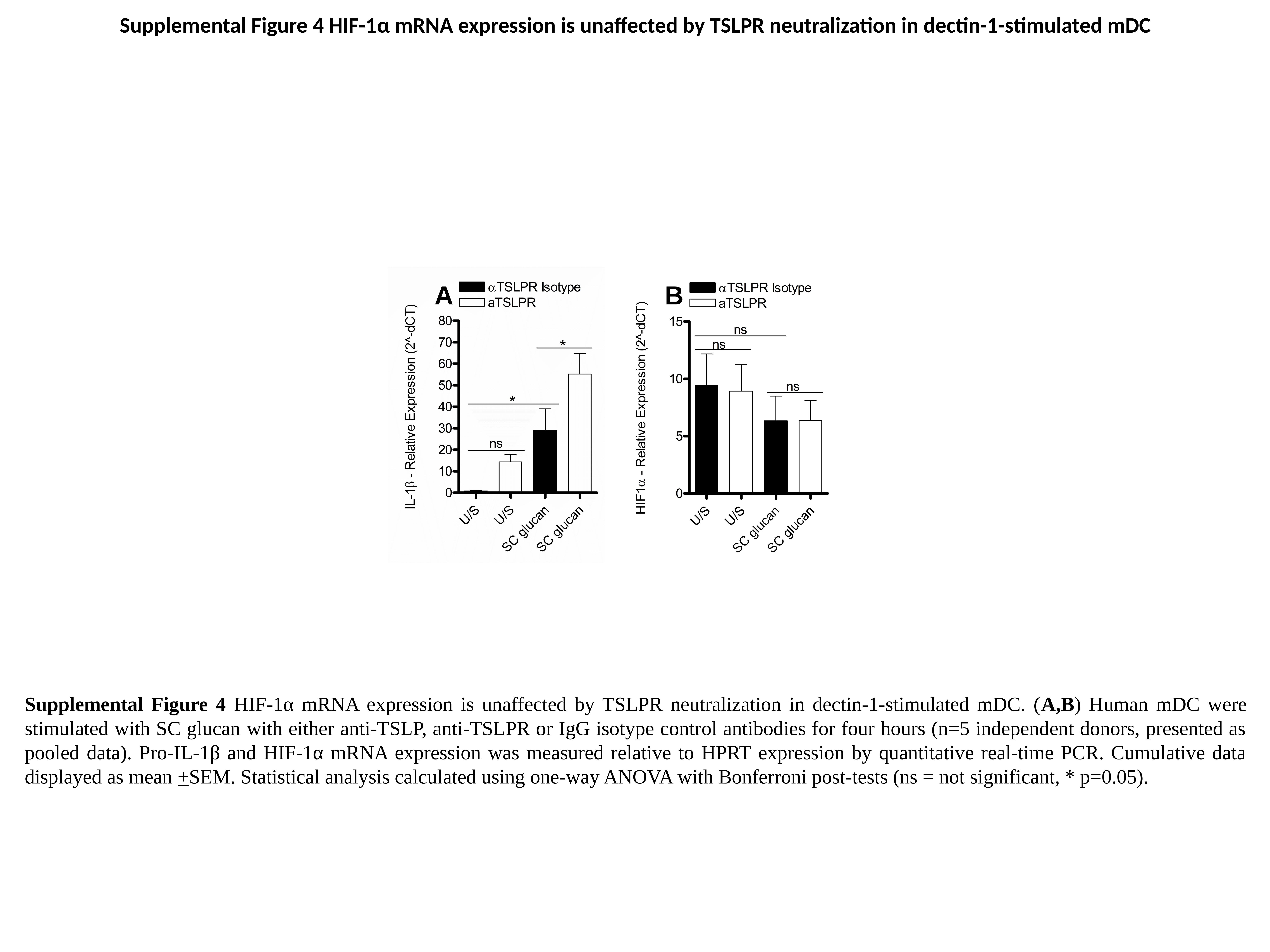

Supplemental Figure 4 HIF-1α mRNA expression is unaffected by TSLPR neutralization in dectin-1-stimulated mDC
A
B
Supplemental Figure 4 HIF-1α mRNA expression is unaffected by TSLPR neutralization in dectin-1-stimulated mDC. (A,B) Human mDC were stimulated with SC glucan with either anti-TSLP, anti-TSLPR or IgG isotype control antibodies for four hours (n=5 independent donors, presented as pooled data). Pro-IL-1β and HIF-1α mRNA expression was measured relative to HPRT expression by quantitative real-time PCR. Cumulative data displayed as mean +SEM. Statistical analysis calculated using one-way ANOVA with Bonferroni post-tests (ns = not significant, * p=0.05).

## Slide 5
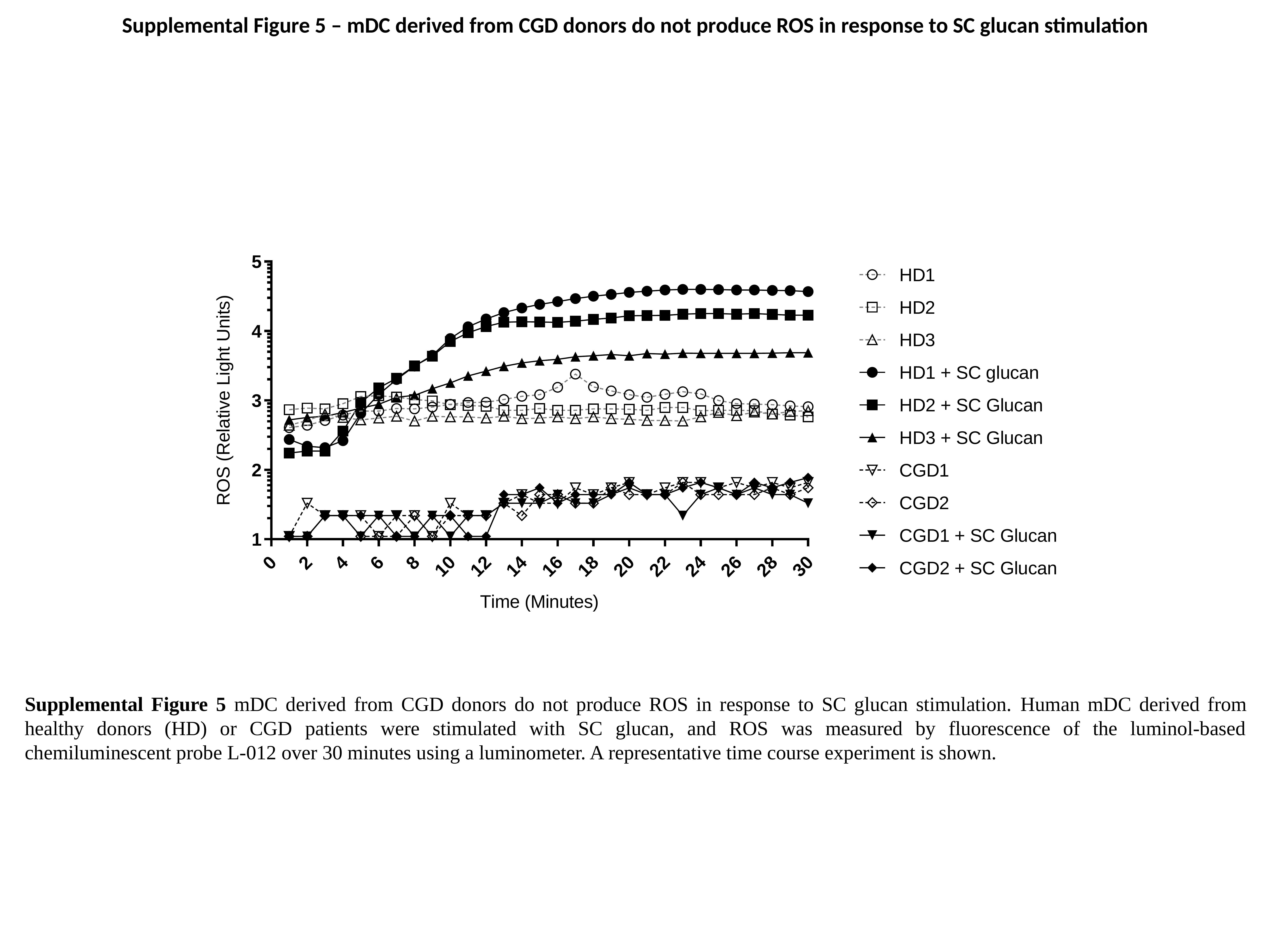

Supplemental Figure 5 – mDC derived from CGD donors do not produce ROS in response to SC glucan stimulation
Supplemental Figure 5 mDC derived from CGD donors do not produce ROS in response to SC glucan stimulation. Human mDC derived from healthy donors (HD) or CGD patients were stimulated with SC glucan, and ROS was measured by fluorescence of the luminol-based chemiluminescent probe L-012 over 30 minutes using a luminometer. A representative time course experiment is shown.

## Slide 6
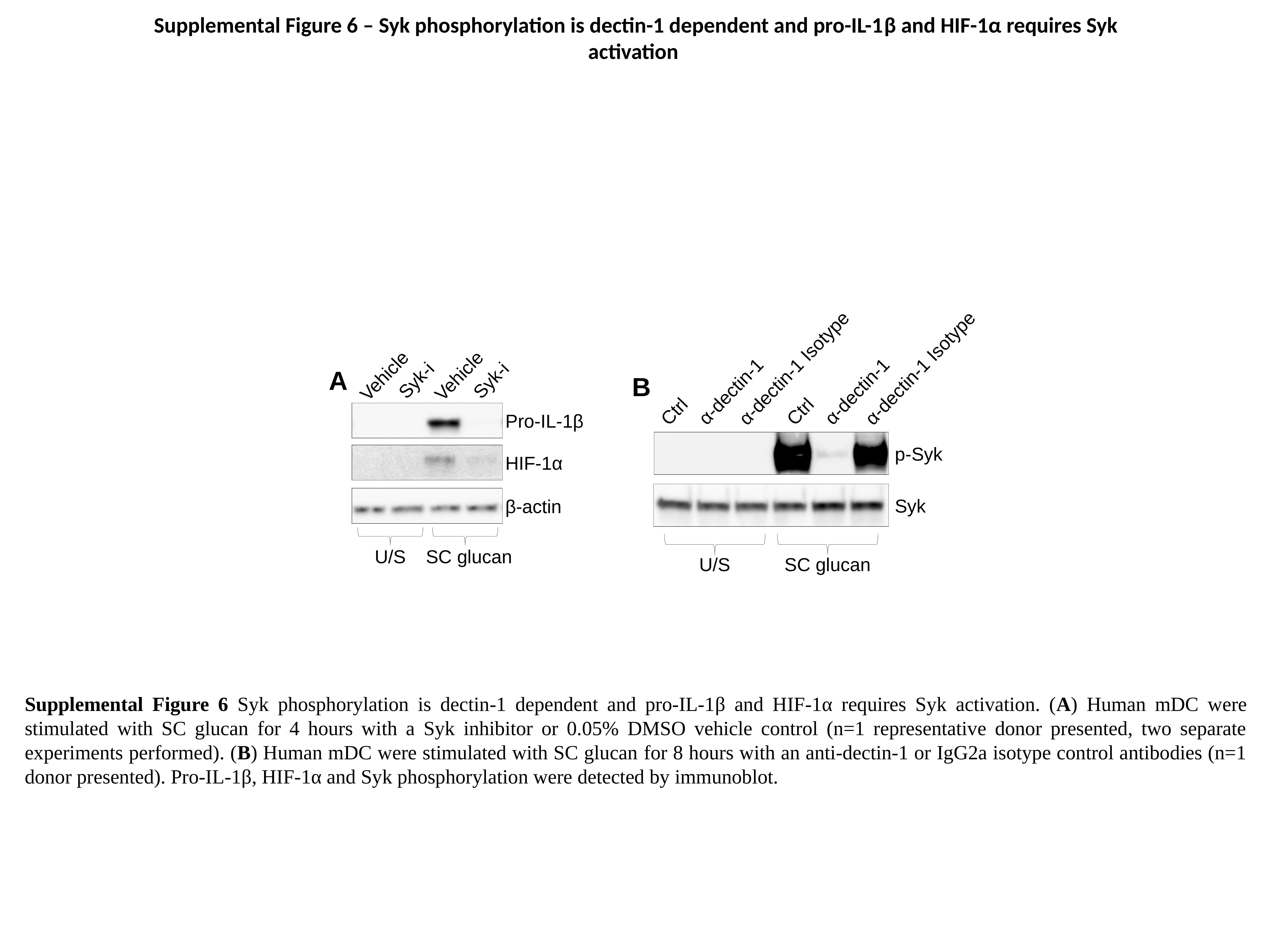

Supplemental Figure 6 – Syk phosphorylation is dectin-1 dependent and pro-IL-1β and HIF-1α requires Syk activation
α-dectin-1 Isotype
α-dectin-1 Isotype
α-dectin-1
α-dectin-1
Ctrl
Ctrl
p-Syk
Syk
U/S
SC glucan
Vehicle
Vehicle
Syk-i
Syk-i
Pro-IL-1β
HIF-1α
β-actin
U/S
SC glucan
A
B
Supplemental Figure 6 Syk phosphorylation is dectin-1 dependent and pro-IL-1β and HIF-1α requires Syk activation. (A) Human mDC were stimulated with SC glucan for 4 hours with a Syk inhibitor or 0.05% DMSO vehicle control (n=1 representative donor presented, two separate experiments performed). (B) Human mDC were stimulated with SC glucan for 8 hours with an anti-dectin-1 or IgG2a isotype control antibodies (n=1 donor presented). Pro-IL-1β, HIF-1α and Syk phosphorylation were detected by immunoblot.
